# Biosorption of Lanthanides from Aqueous Solutions Using Pretreated *Buccinum tenuissimum* Shell Biomass

**DOI:** 10.1155/2010/804854

**Published:** 2010-10-25

**Authors:** Yusuke Koto, Naoki Kano, Yudan Wang, Nobuo Sakamoto, Hiroshi Imaizumi

**Affiliations:** ^1^Graduate School of Science and Technology, Niigata University, Ikarashi 2-nocho 8050, Nishi-Ku, Niigata 950-2181, Japan; ^2^Faculty of Engineering, Niigata University, Ikarashi 2-nocho 8050, Nishi-Ku, Niigata 950-2181, Japan

## Abstract

Biosorption experiment from aqueous solutions containing known amount of rare earth elements (REEs) using pre-treated *Buccinum tenuissimum* shell was explored to evaluate the efficiency of shell biomass as sorbent for REEs. In this work, four kinds of sieved shell samples: (a) “Ground original sample”, (b) “Heat-treatment (480°C, 6 hours) sample”, (c) “Heat-treatment (950°C, 6 hours) sample” and (d) “Heat-treatment (950°C, 6 hours) and water added sample” were used. Furthermore, to confirm the characteristics of the shell biomass, the crystal structure, the surface morphology, and the specific surface area of these shell samples were determined. Consequently, the following matters have been mainly clarified. (1) The crystal structure of the shell biomass was transformed from aragonite (CaCO_3_) into calcite (CaCO_3_) phase by heat-treatment (480°C, 6 hours); then mainly transformed into calcium oxide (CaO) by heat-treatment (950°C, 6 hours), and calcium hydroxide (Ca(OH)_2_) by heat-treatment (950°C, 6 hours) and adding water. (2) The shell biomass showed excellent sorption capacity for lanthanides. (3) Adsorption isotherms using the shell biomass can be described by Langmuir and Freundlich isotherms satisfactorily for lanthanides except “heat-treatment (950°C, 6 hours) sample”. (4) Shell biomass (usually treated as waste material) can be an efficient sorbent for lanthanides in future.

## 1. Introduction

Rare earth elements (REE) have gained considerable attention owing to their unique properties and a wide range of applications [1–4]. These elements and their compounds have found a variety of applications especially in metallurgy, ceramic industry, and nuclear fuel control [5]. For example, current applications of lanthanum as a pure element or in association with other compounds are in super alloys, catalysts, special ceramics, and in organic synthesis [6]. However, the shortage of trace metals including REEs (and the problem of stable supply for these metals) has been concerned in recent years. Therefore, the establishment of the removal or recovery method for trace metals is important from the viewpoint of resources recovery.

Biosorption studies using various low-cost biomasses as adsorbents have been widely performed for the removal of heavy metals from aquatic effluent in large parts of the world recently [7–19]. However, a few reports are available on exploration of marine biomasses [13, 14]. Furthermore, biosorption studies were mainly focused on toxic metals elements as Cd, Pb, As, and Cr for subject elements [9]. 

In our research, the objective elements are mainly rare earth elements (REEs) and uranium (U) from the viewpoint of resources recovery, although REEs do not represent a common toxic threat. In a previous paper [20], we carried out laboratory model experiments for biosorption of REEs and U using seaweed biomass. Moreover, preliminary experiment for biosorption of REEs, Th and U using *Buccinum tenuissimum* shell biomass has been performed recently [21]. Consequently, the following has been mainly obtained: (1) the shell biomass shows excellent sorption capacity for REEs; (2) Adsorption isotherms using the shell biomass can be described by Langmuir and Freundlich isotherms satisfactorily for REEs, but not for Th and U. 

In addition to ground original sample and heat-treatment (480°C, 6 hours) sample used in our preliminary work [21], heat-treatment (950°C, 6 hours) sample and heat-treatment (950°C, 6 hours) and water added sample of *Buccinum tenuissimum* were also used for comparison in present work. Furthermore, the effect of competitive ion on uptake potential of REEs was also studied using original sample to investigate the efficiency of seaweed biomass as sorbent for REEs for more practical use. Among REEs, lanthanides (i.e., La–Lu) were selected in this study, and sorption isotherms of lanthanides were analyzed using Langmuir and Freundlich equations. Moreover, to evaluate the characteristics of the shell biomass used in this work, the crystal structure, the surface morphology, and the specific surface area of the biomass (four kinds of sieved shell samples) were determined by XRD (X-ray powder diffraction), by SEM (Scanning Electron Microscope), and by BET (Brunaeur, Emmet, and Teller) and Langmuir method, respectively.

## 2. Experimental

### 2.1. Reagents and Apparatus

Lanthanides standard solutions used for making the calibration curve were prepared by diluting the multielement standard solutions (XSTC-1 for REEs; 10 mg·dm^−3^ 5% HNO_3_ solution) purchased from SPEX CertiPrep, Inc. (USA). All other chemical reagents were purchased from Kanto Chemical Co., Inc. (Japan). All reagents used were of analytical grade, and water (>18.2 MΩ in electrical resistance) which was treated by an ultrapure water system (Advantec Aquarius: RFU 424TA, Advantec Toyo, Japan) was employed throughout the work. 

An ICP-MS instrument (Agilent HP4500, USA) was employed to determine the concentration of REEs. The operating condition of ICP-MS is shown in Table 1. The specific surface area of biomass sample was measured using Micrometritics TriStar3000 (USA). The BET method and Langmuir method were applied to determine the surface area. Nitrogen (N_2_) gas was used to determine the adsorption isotherms. The crystal structure and the surface morphology of biomass were determined by X-ray powder diffraction (RIGAKU RINT2500HR, Japan) and by SEM (JEOL, JSM-5800, Japan), respectively. The measurement of pH in solution was carried out using a pH meter (HORIBA, F-21, Japan).

### 2.2. Samples

*Buccinum tenuissimum *shellfish samples used for shell biomass in this work were collected at fshermen's cooperative association on the coast of Niigata Prefecture, Japan in June, 2008. After being separated from the meat by boiling, organism shells were washed thoroughly with ultrapure water after being washed with tap water repeatedly. Afterwards, dried overnight in an electric drying oven (Advantec DRA 430DA, Advantec Toyo, Japan) at 60°C, the biomass was ground and sieved through a sieve (SANPO Test Sieves) to remove particles having size more than 600 *μ*m. Afterwards, a part of this sieved materials was heated for 6 hours at 480°C or 950° C in an electric furnace (ISUZU Muffle Furnace STR-14K, Japan). Moreover, adequate ultrapure water was added to a part of heat-treatment (950°C, 6 hours) samples that were heated at 100°C on a hotplate for evaporation to near dryness (removing water) and finally dried in an electric drying oven at 60°C. 

Based on the above-mentioned procedure, four kinds of sieved samples: (a) ground original sample, (b) heat-treatment (480°C, 6 hours) sample, (c) heat-treatment (950°C, 6 hours) sample, and (d) heat-treatment (950°C, 6 hours) and water-added sample have been prepared for sorption experiments of lanthanides in this work. 

### 2.3. Sorption Experiment for Lanthanides Using Seaweed Biomass

The following sorption experiments were performed using *Buccinum tenuissimum* shell biomass. Each sample of 0.2 g was contacted with 100 cm^3^ of multielement standard solution (prepared by XSTC-1) including known initial lanthanide concentration (10 to 500 *μ*g · dm^−3^) in a 200 ml conical flask. Afterwards, the suspensions were shaken for 30 minutes in a water bath at room temperature (25°C) at pH 5. Experimental conditions (i.e., pH, contact time, and biosorbent dose rate) in this work were determined based on our preliminary experiments [21]. The pH of each solution was adjusted by using 0.1 mol·dm^−3^HNO_3_/0.1 mol·dm^−3^ NH_3_aq. Following with each sorption experiment, the suspension containing biomass and lanthanides standard solution was filtered through a 0.10 *μ*m membrane filter (Advantec Mixed Cellulose Ester, 47 mm) to remove lanthanides that have been adsorbed into the shell, and the concentration of these metals in the filtrate was determined with an ICP-MS.

### 2.4. Effect of Competitive Ions on the Sorption of Lanthanides

The effect of competitive ion on the sorption of lanthanides was studied as the following experiment. In this experiment, the initial lanthanides concentration was taken as 100 *μ*g·dm^−3^based on preliminary experiments [21]. In a 200 ml conical flask, each biomass sample (0.2 g) was contacted with 100 cm^3^ of lanthanide solution under the presence of calcium (Ca), magnesium (Mg), sodium (Na), and potassium (K) ion at different concentrations 50, 100, and 200 mg·dm^−3^. Other experimental conditions and methods were basically the same as that mentioned above in Section 2.3. After filtration through a 0.10 *μ*m membrane filter, the matrix in the filtration was removed using chelate disk (47 *φ*mm) (Empore Sumitomo 3M Co.) according to the procedure of Takaku et al. [22], and the concentration of these metals was determined with an ICP-MS.

### 2.5. Adsorption Isotherms

#### 2.5.1. Metal Uptake

The uptake of lanthanides by each sample was calculated using the following mass balance equation [23]:

(1) $$q=\left(C_{o}-C_{e}\right)\frac{V}{W}\left[\text{mg}\cdot {\text{g}}^{\text{-1}}\right],$$

where *q* = metal uptake (mg · g^−1^); *C*_*o*_= initial metal concentration (*μ*g·dm^−3^); *C*_*e*_= final (after sorption at equilibrium) metal concentration (*μ*g·dm^−3^); *V*= volume of the solution (cm^3^); and *W*= dry weight of each sample (g).

Adsorption isotherms of sorption data were studied at varying initial concentration from 10 to 500 *μ*g·dm^−3^ under optimized condition of pH, contact time, and biosorbent dosage in this work. The adsorption data obtained for lanthanides were analyzed using Freundlich and Langmuir equations.

#### 2.5.2. Langmuir and Freundlich Isotherm Model

Two common adsorption model, Langmuir and Freundlich isotherm model was applied based on Dahiya et al. [13, 14] to evaluate the adsorption data obtained in this study. 

Langmuir model assumes monolayer sorption onto a surface and is given by

(2) $$\frac{C_{e}}{q_{e}}=\left(\frac{C_{e}}{a}\right)+\left(\frac{1}{ab}\right),$$

where *C*_*e*_ (mg·dm^−3^) is the concentration of metal ion at equilibrium in the aqueous solution, *q*_*e*_(mg · g^−1^) is the amount of adsorption at equilibrium, a (mg · g^−1^) is the maximum adsorption capacity, and b (dm^3^ · mg^−1^) is the equilibrium adsorption constant (Langmuir constant) [24, 25]. A plot of *C*_*e*_/*q*_*e*_ versus *C*_*e*_ gives a straight line with slope of1/*a*, and intercept is 1/(*a* *b*); *b* can be related to the adsorption free energy ∆*G *_ads_ (J · mol^−1^) by

(3) $$\Delta G_{\text{ads}}=-\text{R}T\ln\;\;\text{b},$$

where R is the gas constant (8.314 J · K^−1^ · mol^−1^), *T* is the absolute temperature (K) at equilibrium.

The Langmuir constant (*b*) can be used to determine the suitability of the adsorbent to adsorbate by using dimensionless parameter, Hall separation factor (*R*_*L*_) [26], which is defined as

(4) $$R_{L}=\left[\frac{1}{\left(1+\text{b}C_{0}\right)}\right],$$

where *C*_0_(mg·dm^−3^) is the initial concentration. The slope of the linearized Langmuir isotherm can be used to interpret the type of sorption using the value of *R*_*L*_ as follows:

 
*R*_*L*_ < 0 unfavorable;
 
*R*_*L*_ > 1 unfavorable;
 
*R*_*L*_ = 1 favorable;
 0 < *R*_*L*_ < 1 favorable;
 
*R*_*L*_ = 0 irreversible.

On the other hand, Freundlich isotherm can also be used to explain adsorption phenomenon as given below:

(5) $$q_{e}=K_{F}C_{e}^{1/n},$$

where *K*_*F*_ and 1/*n* indicate the adsorption capacity and the adsorption intensity of the system, respectively. It is shown that 1/*n* values between 0.1 and 1.0 correspond to beneficial adsorption. The linearized Freundlich model isotherm is represented by

(6) $${\log\;}_{10}\;q_{e}={\log\;}_{10}\;K_{F}+\left(\frac{1}{n}\right){\log\;}_{10}\;C_{e}.$$

A plot of log _10_ *q*_*e*_ versus log _10_ *C*_*e*_ gives a straight line with slope of 1/*n* and intercept islog _10_ *K*_*F*_.

## 3. Results and Discussion

### 3.1. Characteristics of *Buccinum tenuissimum* Shell Biomass

**Figure 1:**
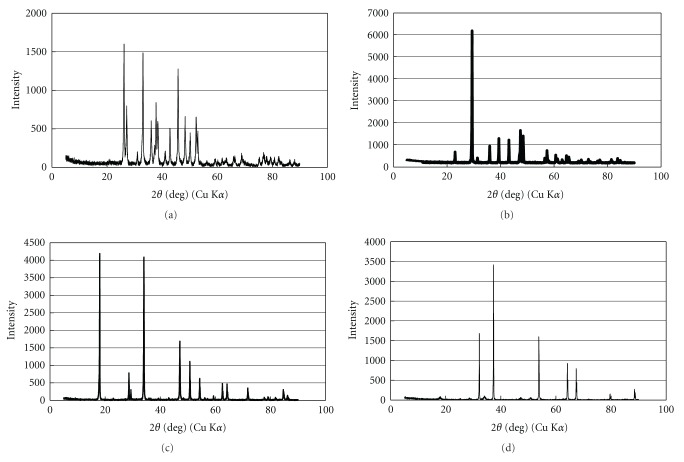
X-ray diffraction (XRD) patterns of *Buccinum tenussimum* shell biomass before adsorption of metals. (a) ground original sample, (b) heat-treatment (480°C) sample, (c) heat-treatment (950°C) sample, and (d) heat-treatment (950°C) and water-added sample.

X-ray powder diffraction (XRD) patterns of the four kinds of *Buccinum tenuissimum* shell biomass samples are shown in Figure 1. The crystal structure of the shell biomass was transformed from aragonite (CaCO_3_) into calcite (CaCO_3_) phase by heat treatment (480°C, 6 hours). Moreover, the crystal structure of the shell biomass was mainly transformed into calcium oxide (CaO) by heat treatment (950°C, 6 hours) and was mainly transformed into calcium hydroxide (Ca(OH)_2_) by adding water after heat treatment (950°C, 6 hours). 

**Figure 2:**
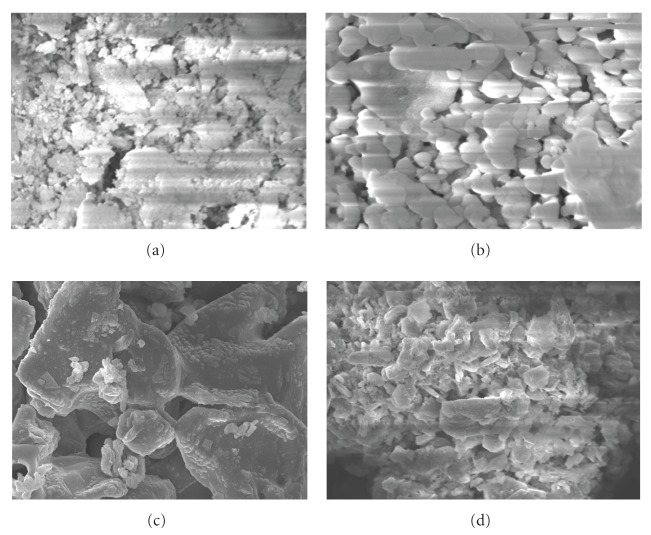
SEM pictures of *Buccinum tenussimum* shell biomass before adsorption of metals. (a) ground original sample, (b) heat-treatment (480°C) sample, (c) heat-treatment (950°C) sample, and (d) heat-treatment (950°C) and water-added sample.

SEM pictures of the four kinds of sieved shell biomass samples: (a) ground original sample, (b) heat-treatment (480°C, 6 hours) sample, (c) heat-treatment (950°C, 6 hours) sample, and (d) heat-treatment (950°C, 6 hours) and water-added sample are shown in Figure 2. Comparing Figure 2(b) with Figure 2(a), comparatively clear crystal with a lot of big particles may be observed by heat treatment (480°C, 6 hours). It is suggested that ground original sample contains a lot of organic materials such as protein, and most of organic matter seem to disappear by heat treatment (480°C, 6 hours). Moreover, fine crystal particle was not observed in (c) heat-treatment (950°C, 6 hours) sample. This may be attributable to the phenomena that many crystals were connected largely with each other due to high-temperature sintering. Meanwhile, relative clear crystal (sizes are mostly 1.0–4.0 *μ*m) was observed in (d) heat-treatment (950°C, 6 hours) and water-added sample. 

Furthermore, the measurement of specific surface area of the four kinds of sieved samples was performed in this study; and the results are shown in Table 2 along with the main crystal structure of these samples. Remarkably, decrease of specific surface area (i.e., from 3.32 m^2^/g to 0.390 m^2^/g for BET, or from 5.35 m^2^/g to 0.612 m^2^/g for Langmuir) was found after heat treatment (480°C, 6 hours). It is suggested that the crystal structure transformation (i.e., from aragonite (CaCO_3_) into calcite (CaCO_3_) phase) and also the difference of the surface morphology can be closely related to the remarkable decrease of specific surface area of the shell biomass. On the other hand, the surface area of heat-treatment (950°C, 6 hours) sample was 1.88 m^2^/g for BET or 3.10 m^2^/g for Langmuir, respectively, and that of “heat-treatment (950°C, 6 hours) and water-added sample” was 6.37 m^2^/g for BET or 9.91 m^2^/g for Langmuir, respectively.

### 3.2. Comparison for Sorption Capacity of Lanthanides by Four Kinds of Sieved Biomass

**Figure 3:**
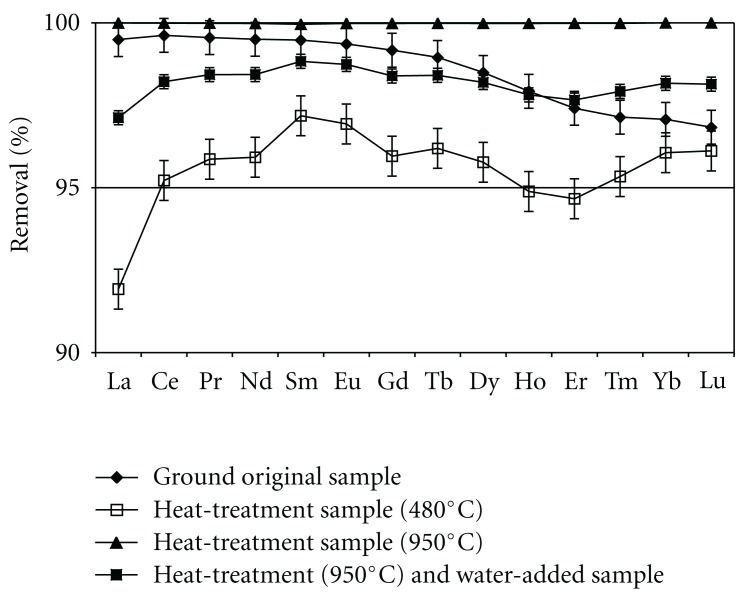
Comparison for sorption capacity of lanthanides by four kinds of sieved *Buccinum tenuissimum* shell samples.

The comparison for sorption capacity of lanthanides by four kinds of sieved* Buccinum tenuissimum *shell samples is shown in Figure 3. In this experiment, the initial lanthanides concentration was taken as 100 *μ*g · dm^−3^. From this figure, it is found that all kinds of sieved samples showed excellent sorption capacity under this experimental condition. However, the sorption capacity in sample (b) (i.e., the main phase is calcite) decreases slightly relative to that of the original material (i.e., (a) the main phase is aragonite) and others. The decrease of sorption capacity in sample (b) may be attributable to the remarkable decrease (i.e., by a factor of less than one eighth) of specific surface area of the biomass. 

Prieto et al. [27] pointed that the sorption capacity of calcite is considerably lower than that of aragonite for Cd. In case of lanthanides, similar tendency of sorption capacity were suggested from our work.

### 3.3. Effect of Competitive Ions on the Sorption of Lanthanides

**Figure 4:**
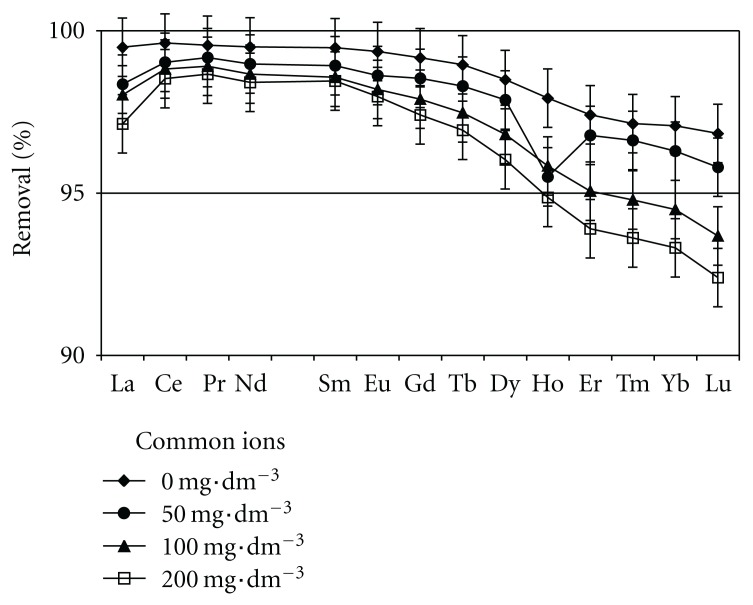
Effect of common ions (Ca^2+^, Mg^2+^, Na^+^, and K^+^) on the removal efficiency of lanthanides using ground original sample.

The percentage removal of REEs under the presence of common ions (Ca^2+^, Mg^2+^, Na^+^, and K^+^) at different concentrations 50, 100, and 200 mg · dm^−3^ is shown in Figure 4. From this figure, the remarkable decrease of sorption capacity of lanthanides was not observed. Even when the concentrations of common ions are 200 mg · dm^−3^, the percentage removal of light REE (LREE) such as La or Ce decreased slightly (2–3%) whereas the removal decreased about 5% for heavy REE (HREE) such as Yb or Lu. This implies that the shell biomass can be an efficient adsorbent for lanthanides in aqueous environment such as seawater, although it requires further investigations to apply the shell biomass to use as an adsorbent for lanthanides more practically.

### 3.4. Characteristics of *Buccinum tenuissimum* Shell Biomass After Adsorption of Metals

**Figure 5:**
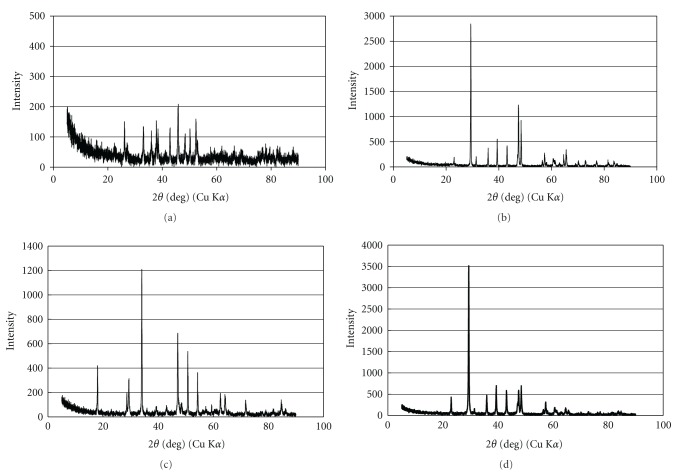
X-ray diffraction (XRD) patterns of *Buccinum tenussimum* shell biomass after adsorption of metals. (a) ground original sample, (b) heat-treatment (480°C) sample, (c) heat-treatment (950°C) sample, and (d) heat-treatment (950°C) and water-added sample.

X-ray diffraction (XRD) patterns of four kinds of sieved samples after adsorption of metals are shown in Figure 5. Similar to the XRD patterns before adsorption of metals (Figure 1), aragonite and calcite were found as the main crystal structure in (a) ground original sample and (b) heat-treatment (480°C, 6 hours) sample, respectively. However, the decrease of peak and increase of noise were also observed in both patterns, particularly in the ground original material as shown in Figure 5(a). Bottcher [28] pointed out that the natural powdered aragonite was transformed to mixed rhombohedral carbonates by the reaction with (Ca, Mg)-chloride solutions. Therefore, there is the possibility that the transformation of aragonite occurred by the reaction with lanthanides in our experiment.

Moreover, according to XRD analysis, the main crystal structure of (c) heat-treatment (950°C) sample was transformed from calcium oxide (CaO) to the mixture of calcium hydroxide (Ca(OH)_2_) and calcite (CaCO_3_) after exposing metals and that of (d) heat-treatment (950°C) and water added sample was transformed from calcium hydroxide(Ca(OH)_2_) to calcite (CaCO_3_) after adsorption of metals. These changes may be due to the reaction with water or carbon dioxide in atmosphere. 

**Figure 6:**
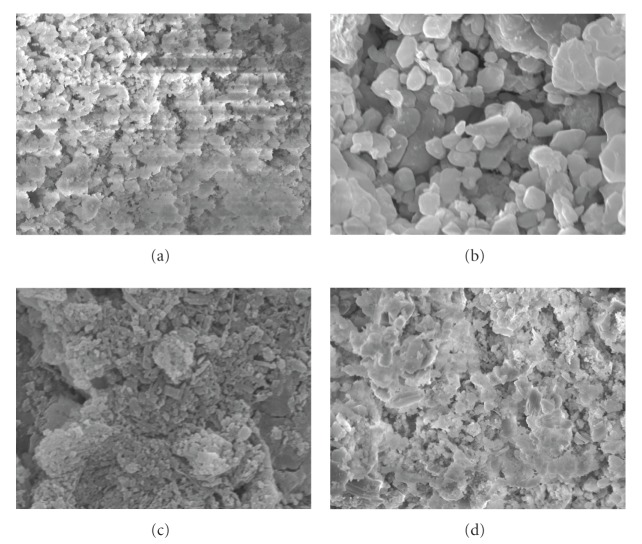
SEM pictures of *Buccinum tenussimum* shell biomass after adsorption of metals. (a) ground original sample, (b) heat-treatment (480°C) sample, (c) heat-treatment (950°C) sample, and (d) heat-treatment (950°C) and water-added sample.

SEM pictures of the shell biomass after adsorption of metals are shown in Figure 6 ((a) ground original sample, (b) heat-treatment sample (480°C), (c) heat-treatment sample (950°C), and (d) heat-treatment and water-added sample (950°C)). By comparing SEM pictures in Figure 2 with that in Figure 6, it is found that the morphology of sample (a) and (b) has hardly changed even after exposing metals. From this observation, these sieved samples should be predicted to withstand the repeated use, and hence it can be a good adsorbent. 

In contrast to sample (a), clear crystal structure (sizes are mostly 0.25–2.0 *μ*m) was observed in sample (b) even after adsorption of metal. In case of Cd conducted by Kohler et al. [29], the difference of procedure for reaction with metals between aragonite and calcite was suggested. According to their work, the precipitation of several distinct types of crystals was observed after exposing metals in the case of aragonite. Then, it is anticipated that similar phenomenon were occurred by adsorption of lanthanides in case of our samples. 

On the other hand, the surfaces of samples (c) and (d) after exposing metals have changed largely compared to that before adsorption of metals (Figure 2). This is in good accord with the results of XRD patterns. Particularly, remarkable transformation was observed in the morphology of sample (d). The reaction of sample (d) with metal solution is supposed to proceed rapidly.

### 3.5. Adsorption Isotherms

The adsorption data obtained for lanthanides using *Buccinum tenuissimum* shell biomass were analyzed using Langmuir and Freundlich equations. 

The correlation coefficient (*R* ^2^) of Langmuir and Freundlich isotherms for lanthanides using ground original shell biomass is shown in Table 3 along with other relevant parameters. From this table, it is found that *R* ^2^ value for lanthanides is comparatively high. It indicates the applicability of these adsorption isotherms satisfactorily for lanthanides in this sample. The dimensionless parameter Hall separation factor (*R*_*L*_) for lanthanides is in the range of 0 < *R*_*L*_ < 1, which means that the sorption for lanthanides by this shell biomass is favorable. Furthermore, the negative value of ∆*G* indicates that the sorption is spontaneous. The higher *R* ^2^ value for Freundlich model rather than for Langmuir isotherm (0.638–0.886 for Langmuir isotherm and 0.844–0.932 for Freundlich one) suggests that the adsorption on this sample is due to multilayer coverage of the adsorbate rather than monolayer coverage on the surface. It is noted that the value of 1/*n* less than unity indicates better adsorption mechanism and formation of relatively stronger bonds between adsorbent and adsorbate [13]. That is to say, favorable adsorption for lanthanides by this shell biomass is presented. 

On the other hand, *R* ^2^ and other parameters of Langmuir and Freundlich isotherms for lanthanides using heat-treatment (480°C) sample is shown in Table 4. It is noteworthy that *R* ^2^ value for REEs in this sample is still more large (0.947–0.982 for Langmuir isotherm and 0.948–0.975 for Freundlich one), compared with the original ground sample (Table 3). Futhermore, this result indicates the stronger the monolayer adsorption (the surface adsorption) on the heat-treatment sample relative to on the original sample (before heat treatment). Judging from the value of *R*_*L*_ or 1/*n* in Table 4, the heat-treatment (480°C) sample also exhibits the favorable property for lanthanides adsorption. 

The correlation coefficient (*R* ^2^) and other parameters of Langmuir and Freundlich isotherms for lanthanides using heat-treatment (950°C) sample is shown in Table 5. It is found that *R^2^* value for lanthanides in this sample is fairly small compared with the values of ground original sample or heat-treatment (480°C) sample (in case of La, Ce, Yb and Lu, *R* ^2^ cannot be estimated due to the lack of sorption data at low initial concentration). The low correlation coefficient (R2) in this heat-treatment (950°C) sample may indicate that the removal of lanthanides occurred not by adsorption mechanism, Particularly *R^2^* value is remarkably small for Langmuir isotherm, and then other relevant parameters cannot be estimated. As for Freundlich one, not only *R* ^2^ value is relatively small (0.157–0.625), but the value of 1/*n* for most lanthanide is more than unity. That is to say, the almost perfect removal of lanthanides for this sample (as shown in Figure 3) may be due to other mechanism rather than the adsorption on the biomass. However, the cause or mechanism of lanthanides removal on this sample has yet to be sufficiently clarified in our work, and further investigation to survey the mechanism is needed. 

Finally, *R* ^2^ and other parameters of Langmuir and Freundlich isotherms for lanthanides using heat-treatment (950°C) and water-added sample is shown in Table 6. It is found that *R* ^2^ value for lanthanides in this sample is fairly large particularly for Langmuir isotherm (0.992–0.999 for Langmuir isotherm and 0.885–0.951 for Freundlich one). This result is similar to that for heat-treatment (480°C) sample and indicates the stronger the monolayer adsorption on this sample. Judging from the value of *R *_L_ or 1/*n* in Table 6, this sample also exhibits the favorable conditions for lanthanides adsorption.

As mentioned above, biosorption studies have been mainly focused on toxic metals elements such as Cd, Pb, As, and Cr so far, and a few reports are focused on lanthanides. Hence, the comparison of maximum adsorption capacity (*q*_*m*_) of the biomass for lanthanides in the present study with that of different biosorbents (for lanthanides and other elements) in the literatures [5, 6, 8, 15–19] are presented in Table 7. As clearly seen in Table 7, the biosorption capacity of *Buccinum tenuissimum* shell biomass for lanthanides in this work is smaller than that of the presented biomasses. However, the sorption experiments in this work were carried out under low concentration of lanthanide (i.e., 100 cm^3^ of multielement standard solution including known initial lanthanide concentration (10 to 500 *μ*g · dm^−3^)) instead of that in single component system.

Then, sorption experiment for three lanthanides (La, Eu and Yb) in single component system is being planned using the solution individually prepared by each nitrate salt: La(NO_3_)_3_ · 6H_2_O, Eu(NO_3_)_3_ · 6H_2_O, or Yb(NO_3_)_3_ · 3H_2_O (supplied by Kishida Kagaku Company, Japan) and this *Buccinum tenuissimum* shell biomass.

## 4. Conclusion

Biosorption characteristic of *Buccinum tenuissimum* shell biomass was studied for lanthanides. Sorption isotherms of lanthanides were analyzed using Langmuir and Freundlich equations to confirm the efficiency of shell biomass as sorbent.

The shell biomass samples showed excellent sorption capacity for lanthanides under this experimental condition, and the effect of all the common ions (Ca^2+^, Mg^2+^, Na^+^, and K^+^) up to the concentration of 200 mg · dm^−3^ on the sorption capacity of lanthanides is considerably small. From these results, it was quantitatively clarified to some extent that shell biomass can be an efficient sorbent for lanthanides. It is very significant information from the viewpoint of environmental protection that the shell (usually treated as waste material) can be converted into a biosorbent for lanthanides.

## Figures and Tables

**Table 1:** Operating conditions of ICP-MS.

RF power	1400 W
Plasma gas flow	15 l · min ^−1^

Carrier gas flow	1.2l · min ^−1^

Sampling depth	6.5 mm

Sample uptake rate	0.5 ml · min ^−1^

Measurement point	3 points/peak

Integration time	1.0 sec/point

Measured Isotope	^139^La, ^140^Ce, ^141^Pr, ^146^Nd, ^147^Sm, ^153^Eu,^ 157^Gd, ^159^Tb, ^163^Dy,^ 165^Ho,^ 166^Er, ^169^Tm, ^172^Yb, ^175^Lu

**Table 2:** The crystal structures and the specific surface areas of four kinds of sieved *Buccinum tenuissimum* shell biomass.

Sample	Main crystal structure	Specific surface area
(a) Ground original sample	Aragonite (CaCO_3_)	3.31 m^2^/g (BET) 5.35 m^2^/g (Langmuir)
(b) Heat-treatment (480°C) sample	Calcite (CaCO_3_)	0.390 m^2^/g (BET) 0.612 m^2^/g (Langmuir)
(c) Heat-treatment (950°C) sample	Lime syn. (CaO)	1.88 m^2^/g (BET) 3.10 m^2^/g (Langmuir)
(d) Heat-treatment (950°C) and water added sample	Portlandite (Ca(OH)_2_)	6.37 m^2^ /g (BET) 9.91 m^2^/g (Langmuir)

**Table 3:** Coefficient of Langmuir and Freundlich isotherms for lanthanides using original Buccinum tenuissimum shell biomass.

	Langmuir isotherm				Freundlich isotherm			
	*a*	*b*	*R*_2_	Δ*G*_ads_/KJmol^−1^	*R*_*L*_	*K*_*F*_	1/*n*	*R*_2_
La	400	0.490	0.638	−15.3	0.0200	115	0.654	0.844
Ce	370	1.17	0.886	−17.5	0.0084	163	0.583	0.864
Pr	400	0.714	0.750	−16.3	0.0138	145	0.658	0.853
Nd	400	0.610	0.681	−15.9	0.0161	133	0.662	0.846
Sm	417	0.6.32	0.740	−16.0	0.0156	145	0.709	0.863
Eu	417	0.571	0.794	−15.7	0.0172	136	0.723	0.883
Gd	435	0.418	0.788	−15.0	0.0234	114	0.765	0.904
Tb	476	0.328	0.800	−14.4	0.0296	105	0.778	0.912
Dy	476	0.239	0.849	−13.8	0.0367	81.8	0.804	0.932
Ho	476	0.183	0.870	−12.9	0.0519	65.2	0.799	0.924
Er	476	0.148	0.878	−12.4	0.0633	55.6	0.787	0.920
Tm	476	0.135	0.842	−12.2	0.0687	52.6	0.785	0.910
Yb	476	0.136	0.818	−12.2	0.0683	53.3	0.762	0.887
Lu	500	0.119	0.786	−11.8	0.0775	51.3	0.759	0.877

**Table 4:** Coefficient of Langmuir and Freundlich isotherms for lanthanides using Buccinum tenuissimum shell biomass after heat treatment (480°*C*, 6 hours).

	Langmuir isotherm				Freundlich isotherm			
	*a*	*b*	*R*_2_	Δ*G*_ads_/kJmol^−1^	*R*_*L*_	*K*_*F*_	1/*n*	*R*_2_
La	192	0.243	0.982	−13.6	0.0395	57.2	0.258	0.948
Ce	278	0.234	0.972	−13.5	0.0410	70.4	0.292	0.956
Pr	303	0.229	0.962	−13.5	0.0418	71.5	0.321	0.955
Nd	313	0.225	0.956	−13.4	0.0425	72.5	0.328	0.954
Sm	345	0.266	0.948	−13.8	0.0362	78.4	0.359	0.962
Eu	345	0.248	0.947	−13.7	0.0388	76.0	0.364	0.963
Gd	303	0.231	0.955	−13.5	0.0415	75.0	0.299	0.954
Tb	323	0.221	0.961	−13.4	0.0432	69.8	0.354	0.968
Dy	323	0.195	0.957	−13.1	0.0488	66.3	0.358	0.964
Ho	294	0.178	0.961	−12.8	0.0532	61.0	0.346	0.960
Er	294	0.171	0.963	−12.7	0.0553	59.1	0.355	0.964
Tm	303	0.176	0.964	−12.8	0.0539	59.0	0.372	0.968
Yb	323	0.181	0.960	−12.9	0.0523	60.8	0.395	0.974
Lu	333	0.176	0.966	−12.8	0.0536	62.4	0.389	0.975

**Table 5:** Coefficient of Langmuir and Freundlich isotherms for lanthanides using Buccinum tenuissimum shell biomass after heat treatment (950°*C*, 6 hours).

	Langmuir				Freundlich			
	*a*	*b*	*R* ^2^	Δ*G*_ads_/kJmol^−1^	*R*_*L*_	*K*_*F*_	1/*n*	*R* ^2^
La	*―*	*―*	*―*	*―*	*―*	*―*	*―*	*―*
Ce	*―*	*―*	*―*	*―*	*―*	*―*	*―*	*―*
Pr	*―*	*―*	0.0553	*―*	*―*	5690	0.999	0.418
Nd	*―*	*―*	0.0727	*―*	*―*	10100	1.27	0.375
Sm	*―*	*―*	0.00190	*―*	*―*	838	0.724	0.157
Eu	*―*	*―*	0.2107	*―*	*―*	41600	1.65	0.521
Gd	*―*	*―*	0.157	*―*	*―*	44300	1.69	0.526
Tb	*―*	*―*	0.0974	*―*	*―*	7980	1.08	0.599
Dy	*―*	*―*	0.108	*―*	*―*	13900	1.32	0.506
Ho	*―*	*―*	0.101	*―*	*―*	11100	1.26	0.529
Er	*―*	*―*	0.110	*―*	*―*	8290	1.14	0.625
Tm	*―*	*―*	0.0915	*―*	*―*	8830	1.15	0.588
Yb	*―*	*―*	*―*	*―*	*―*	*―*	*―*	*―*
Lu	*―*	*―*	*―*	*―*	*―*	*―*	*―*	*―*

*―* stands for no data.

**Table 6:** Coefficient of Langmuir and Freundlich isotherms for lanthanides using Buccinum tenuissimum shell biomass after heat-treatment (950°*C*, 6 hours) and adding water.

	Langmuir				Freundlich			
	*a*	*b*	*R*_2_	Δ*G*_ads_/kJmol^−1^	*R*_*L*_	*K*_*F*_	1/*n*	*R* ^2^
La	161	0.969	0.999	−17.0	0.0102	59.3	0.264	0.951
Ce	200	0.980	0.999	−17.1	0.0101	70.3	0.283	0.950
Pr	217	0.852	0.998	−16.7	0.0116	69.3	0.327	0.919
Nd	222	0.789	0.997	−16.5	0.0125	68.0	0.340	0.930
Sm	233	0.878	0.996	−16.8	0.0113	72.0	0.364	0.937
Eu	233	0.782	0.996	−16.5	0.0126	68.3	0.380	0.917
Gd	227	0.647	0.997	−16.0	0.0152	61.6	0.384	0.937
Tb	227	0.629	0.996	−16.0	0.0157	61.7	0.390	0.937
Dy	233	0.506	0.996	−15.4	0.0194	57.2	0.409	0.936
Ho	227	0.404	0.996	−14.9	0.0242	50.6	0.425	0.931
Er	222	0.372	0.996	−14.7	0.0262	47.5	0.433	0.928
Tm	233	0.352	0.996	−14.5	0.0276	48.0	0.450	0.922
Yb	244	0.398	0.994	−14.8	0.0245	55.3	0.417	0.934
Lu	217	0.495	0.992	−15.4	0.0198	53.2	0.409	0.885

**Table 7:** Comparison of adsorption capacity of the biomass for lanthanides with that of different biosorbents for lanthanides including other elements.

Biosorbent	Element	pH	*q*_m_/mg · g^−1^	Reference
*Portumus sanguinolentus*	La	5	140	[5]
*Platanus orientalis*	La	4	28.7	[6]
*Platanus orientalis*	Ce	4	32.1	[6]
*Sargassum polycystum*	La	5	139	[8]
*Sargassum polycystum*	Eu	5	152	[8]
*Sargassum polycystum*	Yb	5	155	[8]
*Drepanocladus revolvens*	Hg	5.5	94.4	[15]
*Xanthoparmelia conspersa*	Hg	6	82.8	[16]
*Ceramium virgatum*	Cd	5	39.7	[17]
*Ulva lactuca*	Pb	5	34.7	[18]
*Ulva lactuca*	Cd	5	29.2	[18]
*Cladophora hutchinsiae*	Ce	5	74.9	[19]
*Buccinum tenuissimum*	Ln*	5	6.20*	Present study

*Ln stands for lanthanides (La–Lu), and *q*_*m*_ in this table shows the summation of that of each lanthanide
